# Single-incision laparoscopic splenectomy

**DOI:** 10.4103/0972-9941.72385

**Published:** 2011

**Authors:** Manish Joshi, Shrikant Kurhade, M S Peethambaram, Suhas Kalghatgi, Mohan Narsimhan, Ramesh Ardhanari

**Affiliations:** Department of Surgery and Gastroenterology, Meenakshi Mission Hospital and Research Centre, Lake Area, Melur Road, Madurai – 625 107, Tamil Nadu, India

**Keywords:** Single-incision laparoscopic surgery, splenectomy

## Abstract

Single-incision laparoscopic surgery (SILS) is a rapidly evolving field as a bridge between traditional laparoscopic surgery and natural orifice transluminal endoscopic surgery. We present a case of SILS splenectomy performed with conventional laparoscopic instruments in a 7-month-old boy with the diagnosis of multiple splenic abscesses. A 3-cm umbilical incision was used for the placement of two (5 mm) trocars and one 10-mm videoscope (30°). Conventional laparoscopic dissector and grasper were the main tools during surgical procedure. Spleen was removed through the umbilical incision. Although procedures like aingle-incision cholecystectomy have been reported, to the best of our knowledge this is the first report of SILS splenectomy using conventional laparoscopic instruments reported from India and is perhaps the first in an infant in the world literature.

## INTRODUCTION

In general, the benefits of laparoscopic splenectomy in terms of reduced postoperative pain, better cosmesis, shorter hospital stay and convalescence are widely recognised. Current efforts are aimed at further reducing the morbidity associated with minimally invasive surgery. Single-incision laparoscopic surgery (SILS) is presumed to be a step towards pure natural orifice transluminal endoscopic surgery (NOTES).[1] We report one of the initial clinical experiences in India with this new technique. Transumbilical surgery either can be performed with one port having three working channels or three separate trocars introduced through the same umbilical incision. Our institution began performing SILS since January 2009, and subsequently, we developed our technique for SILS with conventional laparoscopic instruments. As far as we know, this is the first case of single-incision laparoscopic splenectomy reported from India.[2]

## CASE REPORT

A 7-month-old male child was admitted under the paediatric service with 2 months history of recurrent fever not associated with rigors. The patient had similar complaints 4 months ago and was treated for upper respiratory infection. There was history of tuberculosis in the family. Clinical examination showed evidence of hepatosplenomegaly. Laboratory parameters including haematology, serum electrolytes, blood cultures, liver function tests, and chest x-ray were unremarkable except for high white cell count. The patient was evaluated and treated with IV antibiotics. However, fever persisted and an ultrasound abdomen scan revealed multiple splenic abscesses. Patient’s parents were counselled and a SILS splenectomy was planned.

### Procedure

**Figure 1:**
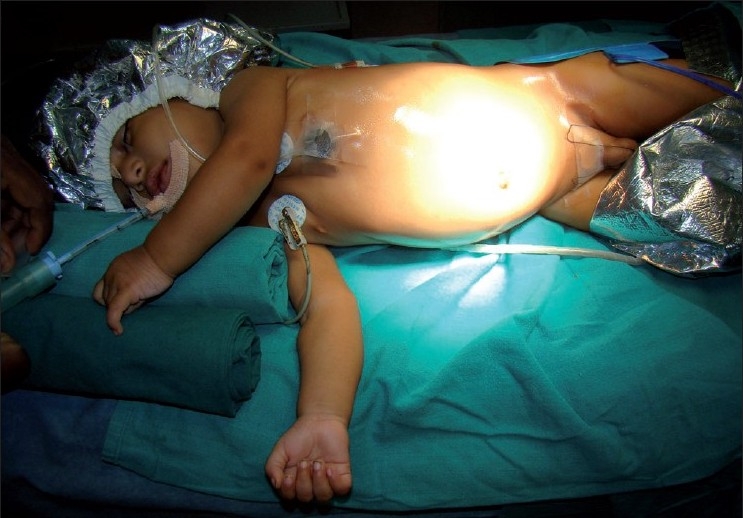
Patient position

**Figure 2:**
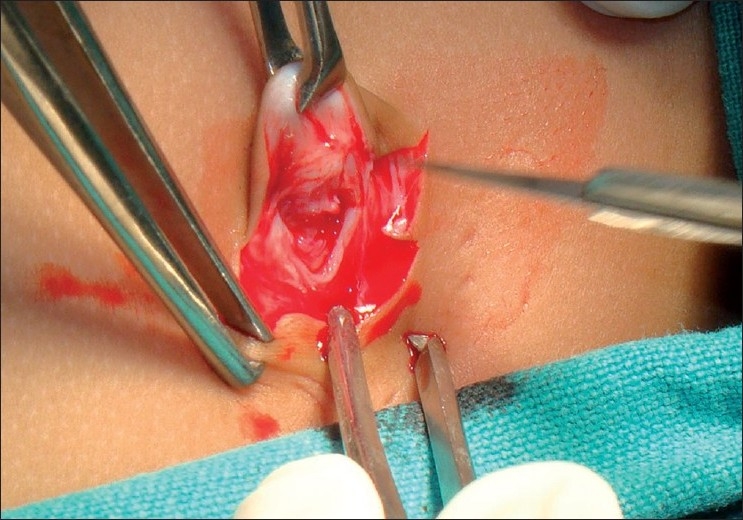
Single transumbilical incision

**Figure 3:**
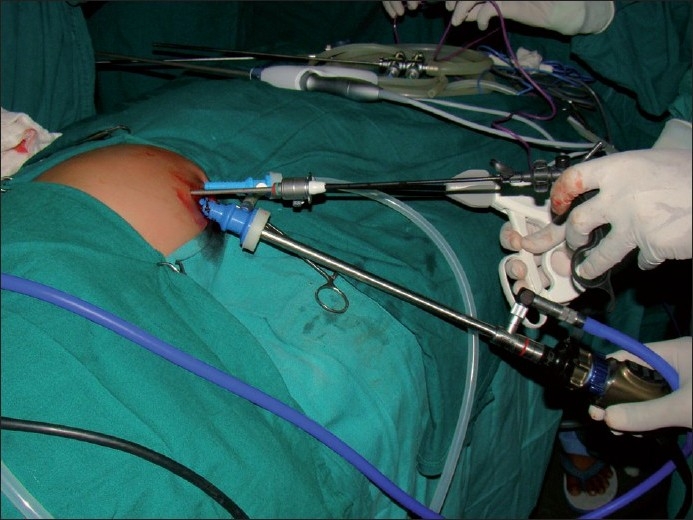
Trocar placements in SILS Splenectomy

**Figure 4:**
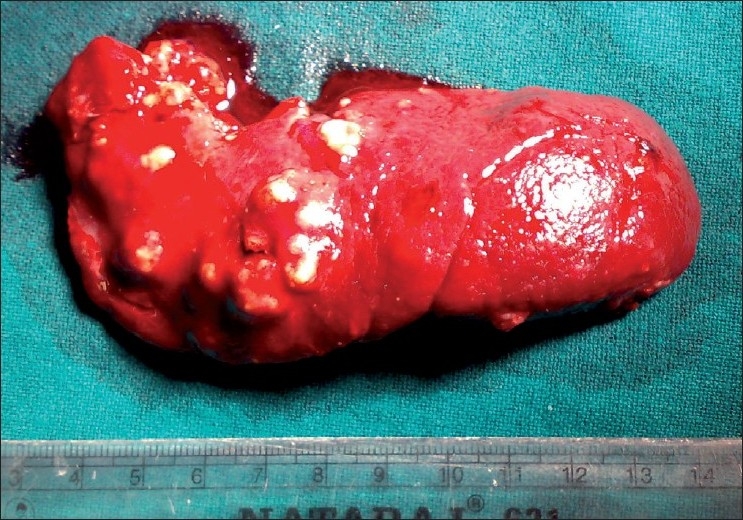
Splenectomy specimen showing multiple abscesses

**Figure 5:**
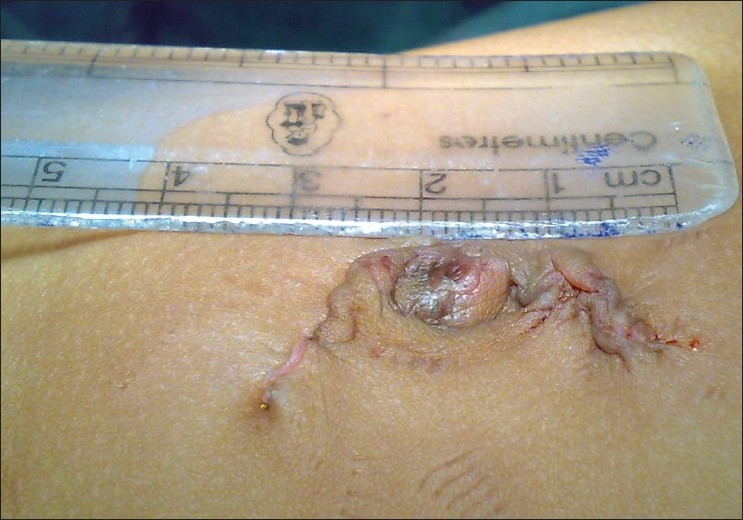
SILS postoperative scar

A surgery was carried out under general anaesthesia. Patient was placed in left upward tilt to 30° [Figure 1]. Open access transumbilical Single-incision of 3 cm was used for placement of three ports (2 × 5 mm, 1 × 10 mm) [Figure 2]. Conventional laparoscopic instruments were used [Figure 3]. The spleen was found plastered to the parietal wall with the hilum exposed, making it convenient to proceed. Splenic attachments were mobilised using harmonic scalpel and hilum was secured with Ligasure (CovidienNorwalk, CT, USA). Two splenenculi were noted and consciously preserved. Splenectomy was done and the specimen retrieved *in toto* through the same single-incision and sent for histopathological examination. Cavity was reinspected and haemostasis confirmed. No drain was placed. The rectus sheath was closed with 2-0 polypropylene. The incision was closed with absorbable subcuticular suture [Figure 4]. The histopathology report showed granulomatous infection [Figure 5]. Patient continued to have postoperative fever for 3 days requiring antibiotics and antipyretics and the symptoms improved. The patient was administered pneumococcal vaccine and started on antitubercular treatment as per paediatrician’s recommendation. The patient was discharged on the fourth postoperative day. Postoperative follow-up at 2 weeks did not reveal any umbilical wound complication.

## DISCUSSION

Laparoscopic splenectomy can be safely performed with a high success rate, low rate of blood loss, and a low rate of perioperative complications.[3] A large single institution series evaluating outcomes in children undergoing laparoscopic splenectomy recently was reported. In a cohort of 223 children undergoing laparoscopic splenectomy for a variety of pathologies, the investigators reported a low morbidity rate (11%) and an impressive early discharge rate, with 70% of children going home on the first postoperative day.[4]

The current paradigm in laparoscopic splenectomy is for each instrument to enter the abdomen through its own separate incision. The quest to make minimally invasive techniques even more “minimal” has generated a drive within the surgical community to explore novel ways of achieving this. This has led to the surgeons either attempting to decrease the number of trocars placed through the abdominal wall or eliminate them completely. This led to the evolution of several approaches, including NOTES and SILS. While NOTES and SILS represent the advent of essentially scarless surgery, there is limited experience. SILS has gained popularity and cholecytectomy has been practiced widely as a formidable alternative to NOTES cholecystectomy. However, the use of SILS in laparoscopic splenectomy is limited to few case reports.[5]

Our institution began performing SILS in January 2009; it started with SILS cholecystectomy and now we use our own technique for SILS with conventional laparoscopic instruments. We recommend the use of conventional laparoscopic instruments to keep the procedure cost same as conventional laparoscopic surgery. It is yet to be proved by trials if SILS requires less analgesia and has better cosmesis. We feel that an added advantage of SILS is that the same incision can be used for retrieval in larger specimens like spleen without the need to extend the incision. The procedure time is not significantly different. The main limitation is that the principle of triangulation in laparoscopic surgery is lost and hence there is a tendency for instruments to clash and fight each other. This we feel is a problem during the learning curve only. We have now switched over to longer length telescope to minimise this problem. The other trick we use is to change the 10-mm telescope to a 5-mm one during retrieval. One needs to transcend the learning curve of SILS by initially performing procedures such as cholecystectomy and then moving on to more complex ones like splenectomy. Though there are case reports from Europe and America regarding SILS splenectomy in children,[6–8] to the best of our knowledge, this is the first of its kind from India and first in the world literature in an infant.

## CONCLUSION

SILS involves performing abdominal operations with laparoscopic instruments placed through a single, small umbilical incision. The primary goal is to avoid visible scarring. SILS splenectomy is safe and feasible in paediatric age group in select patients in expert hands. Technological advancements will further enable SILS.
